# NF2 Inhibits Proliferation and Cancer Stemness in Breast Cancer

**DOI:** 10.1515/med-2020-0042

**Published:** 2020-04-17

**Authors:** Zhibao Wang, Zhiqiang Zhou, Zhe Wang, Yijie Cui

**Affiliations:** 1 Department of Radiology, The No.2 Hospital of Baoding, No. 338 Dongfeng West Road, Jingxiu District, BaoDing 071051, P.R.China

**Keywords:** NF2, breast cancer, stemness, proliferation

## Abstract

**Background:**

Previous studies have shown that NF2 plays a key role in tumorigenesis. NF2 has been illustrated to be downregulated in several types of human cancer. However, the role of NF2 in breast cancer remains unclear.

**Methods:**

We used UALCAN and KM-plotter database to study NF2 expression in human breast cancer and corresponding normal tissues and analyzed its relationship with clinicopathological parameters. We investigated the role of NF2 in breast cancer cells behavior by inhibiting its expression in MDA-MB-231 and MCF-7 cells.

**Results:**

In this study, we found that NF2 was downregulated in breast cancer tissues compared to the adjacent normal tissues. We found that the low expression of NF2 was related with the tumor stage. NF2 overexpression inhibited the cell colon formation and stemness.

**Conclusion:**

Our results indicate a role of NF2 in the progression of breast cancer.

## 1 Introduction

Breast cancer is one of the most common cancers in the world [1]. Although the early onset of breast cancer can be effectively treated, the prognosis of patients with recurrent or distant metastatic breast cancer is poor [2]. Therefore, identifying proteins involved in breast tumorigenesis is very important and can be used as a target for therapeutic or diagnostic strategies.

The NF2 gene has two transcripts of NF2-1 and -2, which are composed of exons 1-15 and 17, and exons 1-15 and 16 respectively. The NF2-1 transcript contains 595 amino acid residues and NF2-2 transcript contains 590 amino acid residues [3]. Sherman et al reported that NF2-17 can prevent the growth of murine schwannoma cells in vitro and in vivo, but NF2-16 does not, so it appears as a tumor suppressor [4]. NF2-17 and NF2-16 are expressed in all tissues and many tumors. Mutations in the NF2 gene can simultaneously affect the transcription of the NF2-1 and NF2-2 subtypes [5]. Studies have shown that the NF2 amino acid sequence is highly homologous to the ERM family proteins, and is collectively referred to as MERM [6]. NF2 consists of three domains, the N-terminal functional region, also known as the FERM domain, which is a highly conserved region of the ERM family [7]. NF2 lacks the actin-binding domain in the ERM protein structure. Both NF2 and ERM proteins are connected to muscle in polarized cells [8]. However, the obvious difference between NF2 and other members of ERM is that it can inhibit tumor growth.

The classical NF2 protein is characterized by benign tumors of the nervous system, and its hallmark symptoms include bilateral vestibular schwannomas, meningioma, and ependymoma [9]. NF2 protein regulates contact-dependent growth inhibition by a signal transduction pathway that controls cell proliferation and survival. The NF2 protein is also a cytoskeleton between the molecular scaffold receptor and cortical actin across the membrane, thereby regulating cell adhesion and migration [10].

Studies have shown that NF2 is a novel tumor suppressor protein, and mutation or loss of expression of NF2 protein leads to tumorigenesis, although its molecular mechanism is not fully understood. Overexpression of NF2 prevents cell proliferation and inhibits oncogene-induced transformation. McClatchey et al. reported that NF2 inhibits proliferation of mitotic signaling and contact-dependent inhibition [11]. Shaw et al. observed that pal active kinase 1 (PAK1) promotes the Cyclin D1 promoter activity, but NF2 inhibits PAK1 activation and prevents Rac from binding and activating PAK1 down-regulated Cyclin D1 expression and inhibiting cell growth [12]. NF2 damages the inhibition effect of proto-oncogene MDM2 on p53 and synergizes with p53 to inhibit tumorigenesis. The 130 amino acid residues at the N-terminus of NF2 may play an important role in the degradation of MDM2, and also increase the transcriptional activity dependent on p53 [13].

However, the expression level and function of NF2 in breast cancer remain unclear. In this study, we found that the expression of NF2 in breast cancer tissues was positively correlated with the tumor prognosis. NF2 over-expression can inhibite the cell colon formation and stemness

## 2 Methods

### 2.1 Cells and reagents

The breast cancer cell lines MDA-MB-231 and MCF-7 were purchased from the American Type Culture Collection (Manassas, VA, USA). Cell lines were cultured in DMEM medium containing 10% fetal bovine serum (Gibco, USA). All cells were incubated at 37°C in a 5% CO2 incubator.

### 2.2 Western blot analysis

To prepare protein extracts, MDA-MB-231 and MCF-7 cells were scraped on the ice, collected by centrifugation (1,2000 x g, 10 min, 4˚C) and incubated with freshly prepared RIPA lysis for 15 min. After centrifugation, the concentration of the protein sample is determined by BCA kit. The protein sample is mixed with the loading buffer and boiled for 8 minutes. After that, the sample was separated by a 10% SDS-PAGE gel and electrotransfered to PVDF membrane. The membrane was blocked in 5% skimmed milk. Subsequently, the membrane was incubated with primary antibodies overnight at 4˚C, followed by secondary antibody. Protein bands were visualized using the ECL prime™ blotting system (GE Healthcare, Little Chalfont, UK).

### 2.3 Proliferation assay

Digestion Cells were harvested on the second day of transfection and inoculated into 96-well plates at a density of 5000 cells/well in triplicate. Subsequently, cultivation for 0, 1, 2, 3 or 4 days, cells were subjected to the CCK8 assay. Briefly, after adding 10μl CCK8 solution to each well, the plate was incubated at 37°C for 4 hours. The absorbance of each well at 595 nm was determined using a microplate reader. The experiment was repeated 3 times.

### 2.4 Colony formation assay

The cells were harvested the next day after transfection. The cells were seeded at a density of 500 cells/well onto six well plates and colonies were formed within 15 days. The cell colonies were fixed in 4% paraformaldehyde and stained with crystal violet. Colonies were counted using a microscope (> 50 cells) directly on the plate. The experiment was repeated 3 times.

### 2.5 Colony formation in soft agarose

For the soft agar colony formation assay, cells were seeded at 0.35% top agarose (Promega) at a density of 500 cells per well, and a 0.7% agarose base was added and complete medium was added. The culture was incubated for 3 wk at 37°C in a humidified incubator. The cells were then stained with 0.5 mL of 0.0005% crystal violet and the colonies were visually counted. All experiments were performed in triplicate.

### 2.6 Cell sphere formation

500 cells were plated in 6-well ultralow attachment plates (3471, Corning) with serum-free medium with 50 ng/ml epidermal growth factor (EGF) (236-EG-200, R&D), 20 ng/ml of basic fibroblast growth factor (bFGF) (233-FB-025, R&D), 1% N2 (17502048, Gibco), 2% B27 (12587-010, Gibco). Then, spheres of diameter greater than 100 μm in each well were counted under a microscope after 10 days.

## 3 Results

### 3.1 Protein expression of NF2 in breast cancer

**Figure 1:**
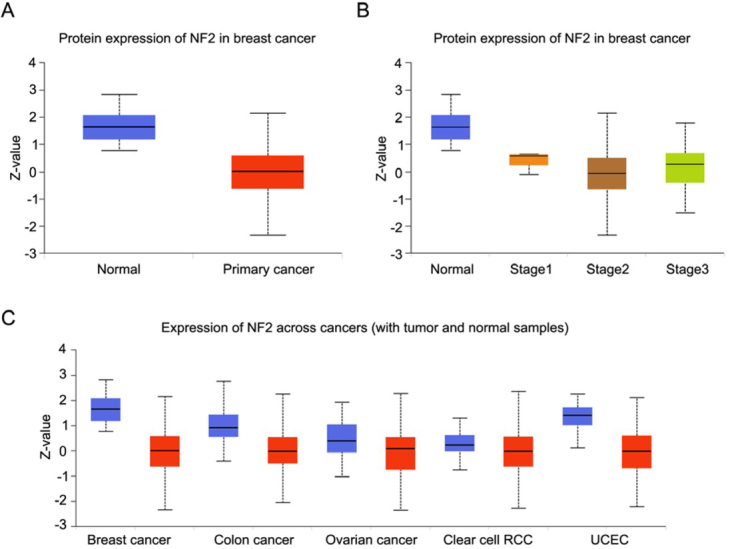
A Protein expression of NF2 in breast cancer. B Protein expression of NF2 in breast cancer based on individual cancer stages. C Pan-cancer view of NF2 expression level.

We performed an analysis about NF2 expression in breast cancer tissues by using UALCAN database (http://ualcan.path.uab.edu) Statistical analysis in the UALCAN database revealed that NF2 was down-expressed in breast cancer compared with the normal group, and the difference was statistically significant (P<0.05), as shown in Figure 1.A. Further analysis found that expression of NF2 was lower in breast cancer based on individual cancer stages than normal tissues, and the difference was statistically significant (P<0.05), as shown in Figure 1.B. To further clarify the relationship between NF2 expression level and cancer, pan-cancer analysis revealed that NF2 has lower expression level in cancer tissue compared with normal tissue, as shown in Figure 1.C.

### 3.2 NF2 is correlated with prognosis

**Figure 2:**
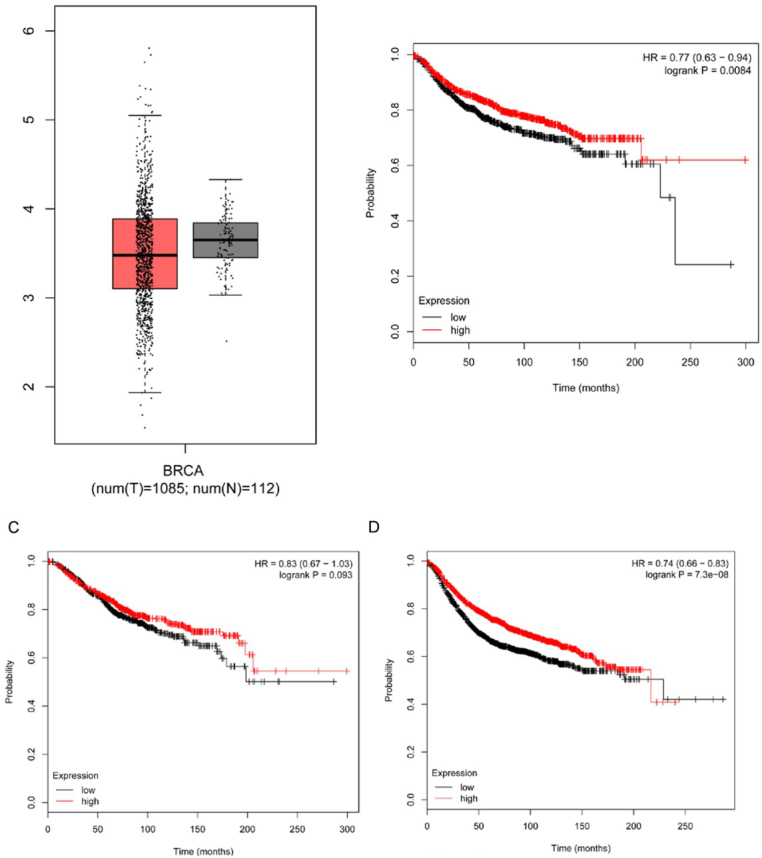
A NF2 mRNA is down-regulated in breast cancer tissues compared to normal tissues in TCGA cohort, analyzed with the Gene Expression Profiling Interactive Analysis (GEPIA). B Relationship between NF2 expression level and distance metastasis free survival of patients with breast cancer. C Relationship between NF2 expression level and overall survival of patients with breast cancer. D Relationship between NF2 expression level and relapse free survival of patients with breast cancer.

We analyzed NF2 mRNA expression in breast cancer tissue by using TCGA database. The statistical analysis shows that NF2 mRNA was downregulated in breast cancer (Figure 2. A). To further clarify the relationship between NF2 expression level and the prognosis of patients with breast cancer, the KM Plotter database analysis was used to show two survival curves of patients with high CD276 expression and low expression group (different probe), and ir was found that CD276 expression level has a significant impact on survival without distant metastasis and recurrence-free survival of patients (Figure 2.B, D). However, for overall survival, that has no significant statistical difference (Figure 2.C).

### 3.3 NF2 inhibits cell colony formation

**Figure 3:**
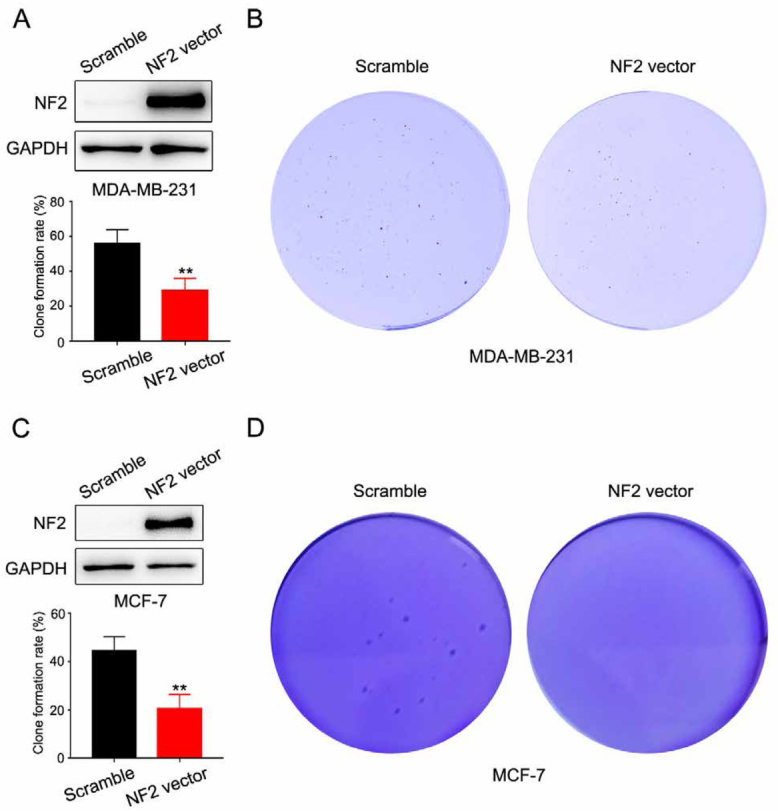
A Western blot analysis of the overexpression of NF2 in MDA-MB-231 cells (up). Clone formation rate of scramble and NF2 vector transfected MDA-MB-231 cells (down). B Representative images of MDA-MB-231 cell colonies in the colony formation assay. C Western blot analysis of the overexpression of NF2 in MCF-7 cells (up). Clone formation rate of scramble and NF2 vector transfected MCF-7 cells (down). D Representative images of MCF-7 cell colonies in the colony formation assay.

We transfected pcDNA3.1-NF2 plasmid into breast cells. We performed a colony formation assay to detect cell proliferation. Compared to the negative control group, over-expression of NF2 obviously suppressed cell proliferation in MDA-MB-231 cells (Figure 3. A and B). We performed colony formation ability in soft agarose. The results showed overexpression of NF2 reduced colony numbers in A549 cells (Figure 3. C and D).

### 3.4 NF2 inhibits cell proliferation and stemness

**Figure 4:**
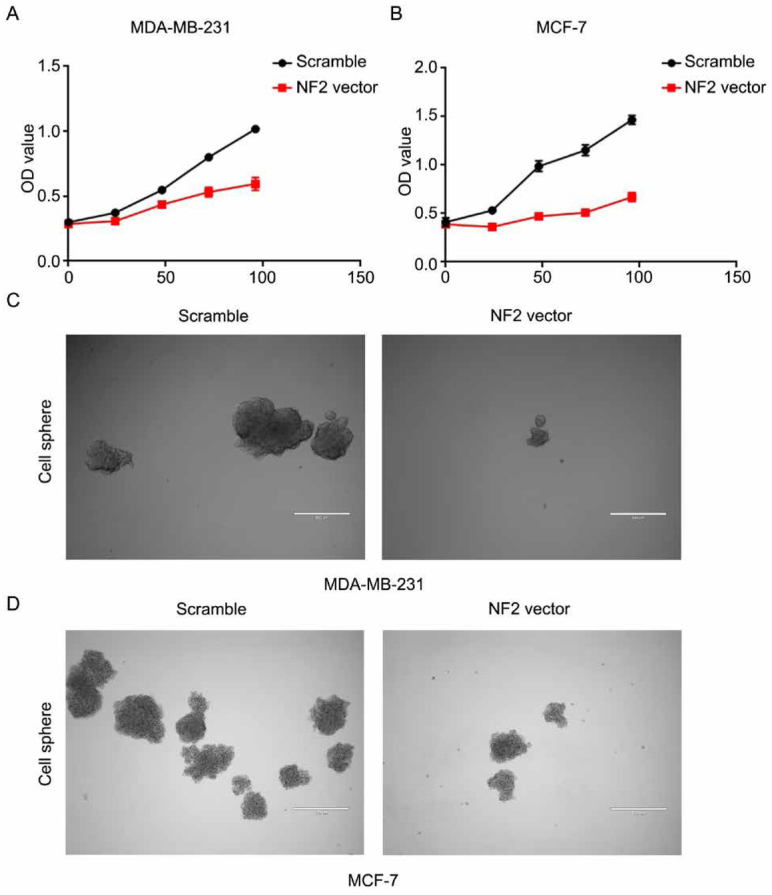
A Proliferation curves of scramble and NF2 vector transfected MDA-MB-231 cells. B Proliferation curves of scramble and NF2 vector transfected MCF-7 cells. C Representative images of in vitro sphere-formation assay of NF2 overexpression in MDA-MB-231 cell and control cell. D Representative images of in vitro sphere-formation assay of NF2 overexpression in MCF-7 cell and control cell.

To explore the impact of NF2 on breast cancer cell proliferation, we performed a CCK8 assay. As shown in Figure 4. A and B, NF2-overexpressed MDA-MB-231 and MCF-7 cells showed weaker proliferation ability compared to negative control siRNA-transfected cells (P < 0.05). The cell sphere assay showed that the overexpression of NF2 suppressed significantly the stemness of MDA-MB-231 and MCF-7 cells (Figure 4.C and D).

## 4 Discussion

At present, there are many studies on NF2 protein in nervous system tumors. Rving et al found that NF2 gene mutations are mostly small fragment gene deletion or insertion, allelic loss, missense mutation, nonsense mutation, etc. Complete deletion of exon 4 is also a common type of mutation [14]. Buceoliero et al observed that the expression of NF2 gene in grade I and II dural sarcoma was higher than that of all dural sarcoma, while the expression of grade III dural sarcoma was lower than the mean [15]. However, inactivation or mutation of the neurofibrin 2 gene (NF2) has not attracted attention in cancer, because they are not common in common human malignancies. But, if they exist, they can affect disease progression, aggressiveness and prognosis. For example, in prostate cancer, inactivation of NF2 is associated with increased invasiveness and chemoresistance [16, 17].

Hiro-kawa et al. found that NF2 directly inhibits the Rac/CDC42-dependent silk/threonine kinase PAK1, which is essential for Ras transformation and NF1 [18]. The mutated NF2 lacks 78 amino acids in the PAK1 inhibitory region and does not inhibit Ras transformation. PAK1-specific inhibitors CEP-1347 and WR-PAK18 selectively inhibited NF2-deficient tumor cells and did not contribute to NF2-positive tumor cells [18]. It indicates that PAK1 is essential for the malignant growth of NF2-deficient cells, and the preventive drug of PAK1 may become a frontier treatment for NF2. These studies suggest that NF2 is closely related to the treatment of tumors.

The NF2 gene is involved in a variety of human tumors and may be a tumor suppressor gene, but its function and mechanism of action are not well understood. An in-depth study of the role of merlin in tumors and its relationship with various signaling molecules and signal transduction pathways may provide an effective biological target for anti-tumor therapy. Activation of NF2 expression may promote tumor cell apoptosis, inhibit tumor growth and metastasis. The use of RNA interference technology to increase NF2 expression may be the goal of treating tumors. However, recent studies suggest that NF2 may also be an oncoprotein. NF2 is expressed differently in different tumors and may be organ specific.

In summary, we investigated the role of NF2 in breast cancer cell stemness, proliferation. Our findings suggest that NF2 may be novel targets for breast cancer therapeutics.
